# Effect of sotatercept on circulating proteomics in pulmonary arterial hypertension

**DOI:** 10.1183/13993003.01483-2024

**Published:** 2024-10-31

**Authors:** Laurent Savale, Ly Tu, Corinne Normand, Athénaïs Boucly, Olivier Sitbon, David Montani, Karen M. Olsson, Da-Hee Park, Jan Fuge, Jan C. Kamp, Marc Humbert, Marius M. Hoeper, Christophe Guignabert

**Affiliations:** 1 Université Paris-Saclay, Hypertension Pulmonaire: Physiopathology and Innovation Thérapeutique, HPPIT, Faculté de Médecine, Le Kremlin-Bicêtre, France; 2 INSERM UMR_S 999, HPPIT, Le Kremlin-Bicêtre, France; 3 Department of Respiratory and Intensive Care Medicine, Assistance Publique Hôpitaux de Paris, Hôpital Bicêtre, ERN-LUNG, Le Kremlin-Bicêtre, France; 4 Department for Respiratory Medicine and Infectious Diseases and German Centre of Lung Research (DZL), Hannover Medical School, Hannover, Germany

## Abstract

Alterations in specific signalling pathways within the bone morphogenetic protein/transforming growth factor-β (BMP/TGF-β) family, involving several precisely regulated activator or inhibitor ligands, have been identified as pathogenic drivers of pulmonary arterial hypertension (PAH). These alterations, particularly affecting BMPRII and activin-dependent pathways, have led to innovative therapies, notably the development of sotatercept [1, 2]. Sotatercept, a fusion protein of the extracellular domain of human ACTRIIA and the Fc domain of human IgG1, has shown promising results in improving key clinical, functional, and haemodynamic parameters in PAH patients, as evidenced by positive results in the phase 2 PULSAR and phase 3 STELLAR trials [3, 4]. This progress was partly based on preclinical studies showing that reducing activin-induced Smad2/3 phosphorylation levels, by suppressing activin production in mice [5] or using soluble receptors in rats [6, 7], can attenuate pulmonary vascular remodelling. Despite these advancements, the precise mechanisms of action of these approaches in humans and rodents need to be better understood to enhance these valuable tools. Sotatercept raises several critical questions regarding its mechanism of action, and a deeper understanding could reveal the pathophysiological mechanisms of PAH, leading to more effective therapeutic approaches.

## Shareable abstract

**Proteomic analysis of circulating biomarkers reveals that sotatercept's impact extends beyond activins to influence BMP-9 and BMP-10, along with essential metabolic and inflammatory factors** https://bit.ly/3Z5AZJ3

*To the Editor*:

Alterations in specific signalling pathways within the bone morphogenetic protein/transforming growth factor-β (BMP/TGF-β) family, involving several precisely regulated activator or inhibitor ligands, have been identified as pathogenic drivers of pulmonary arterial hypertension (PAH). These alterations, particularly affecting BMPRII and activin-dependent pathways, have led to innovative therapies, notably the development of sotatercept [1, 2]. Sotatercept, a fusion protein of the extracellular domain of human ACTRIIA and the Fc domain of human IgG1, has shown promising results in improving key clinical, functional, and haemodynamic parameters in PAH patients, as evidenced by positive results in the phase 2 PULSAR and phase 3 STELLAR trials [3, 4]. This progress was partly based on preclinical studies showing that reducing activin-induced Smad2/3 phosphorylation levels, by suppressing activin production in mice [5] or using soluble receptors in rats [6, 7], can attenuate pulmonary vascular remodelling. Despite these advancements, the precise mechanisms of action of these approaches in humans and rodents need to be better understood to enhance these valuable tools. Sotatercept raises several critical questions regarding its mechanism of action, and a deeper understanding could reveal the pathophysiological mechanisms of PAH, leading to more effective therapeutic approaches.

A total of 31 participants from the PULSAR phase 2 trial (n=7) and the STELLAR phase 3 trial (n=24) at Hannover Medical School, Hannover, Germany were included in this biological study. Among them, 17 were in the placebo group and 14 in the sotatercept group. All participants underwent sample collection at baseline and again 24 weeks later during the follow-up visit. The study received institutional review board approval for both trials, and all patients provided written informed consent. Additionally, all patients signed the broad consent form from the German Centre of Lung Research (https://dzl.de/en/dzl-data-warehouse) approved by the ethics committee (8540_BO_K_2019). For evaluating differences in continuous variables, an independent t-test was used for normally distributed variables, while the Mann–Whitney U-test was applied for non-normally distributed variables. Comparisons of haemoglobin (Hb) levels at baseline and follow-up were conducted using a paired t-test. Measurements of 2554 circulating proteins were performed using the Olink Explore 3072 platform. An unbiased and blinded biostatistical analysis was conducted for each protein using a linear mixed effects model.

The mean±sd age of the study population was 46±13 years, with 25 (81%) being female, and no significant differences between the sotatercept and placebo groups. 19 patients (61%) had idiopathic PAH, eight (26%) heritable PAH, three (10%) repaired congenital heart disease-associated PAH, and one drug-associated PAH. Significant improvements were observed with sotatercept treatment compared to placebo at week 24. Median changes from baseline in the 6-min walk distance were +87 m (interquartile range (IQR) +14 to +115 m) for the sotatercept group *versus* −20 m (IQR −55 to +1 m) for the placebo group (p<0.0001). Pulmonary vascular resistance decreased by −2.7 WU (IQR −6.8 to −1.2 WU) in the sotatercept group, compared to an increase of +0.6 WU (IQR −0.5 to +2.0 WU) in the placebo group (p<0.0001). Mean pulmonary arterial pressure decreased by −16 mmHg (IQR −28 to −9 mmHg) in the sotatercept group, *versus* no change in the placebo group (+0.0 mmHg; IQR −5 to +6 mmHg) (p<0.0001). Additionally, six patients (43%) in the sotatercept group improved in World Health Organization functional class compared to two patients (12%) in the placebo group (p=0.04). In the sotatercept group, seven patients (50%) developed telangiectasia. Patients receiving sotatercept also showed significant increase in Hb levels from 14.5±1.2 g·dL^−1^ at inclusion to 15.8±2.0 g·dL^−1^ at week-24 (p=0.003) without significant change in the placebo group.

We identified 52 proteins with differential expression changes at week 24 between the sotatercept and placebo groups (figure 1a). Notably, reductions in N-terminal pro-brain natriuretic peptide, brain natriuretic peptide, activin, and FSTL3 were observed. Significant reductions in circulating BMP-9 (fold change (FC): −0.28, p*-*adjusted: 1.15×10^−3^) and BMP-10 (FC: −0.63, p-adjusted: 7.98×10^−9^) in sotatercept-treated patients were also noted (figure 1b). This reduction was accompanied by significant changes in proteins associated with inflammation and immune activation, including elevations in E-selectin, macrophage scavenger receptor 1 (MSR1), mannose receptor C-type 1 (MRC1), haem oxygenase 1 (HMOX1), interleukin (IL)-18, and macrophage receptor with collagenous structure (MARCO). Conversely, reductions were seen in C-C motif chemokine ligand-18 (CCL18), and γ-glutamyltransferase (GGT1) (figure 1a). Furthermore, alterations were observed in proteins involved in oxidative stress, cardiovascular integrity, lipid metabolism, amino acid metabolism, and cell cycle regulation (figure 1c). Given the association between telangiectasia and the ALK1/endoglin pathway, we explored potential correlations with serum levels of BMP-9, BMP-10, and soluble forms of ALK1 and endoglin. Among the 7 patients with telangiectasia, only baseline levels of soluble ALK1 were lower compared to those without this adverse effect (0.33±0.21 *versus* 0.68±0.20 normalised protein expression; p=0.07). However, by week 24, there were no significant differences observed between the groups (0.17±0.13 *versus* 0.87±0.16).

**FIGURE 1:**
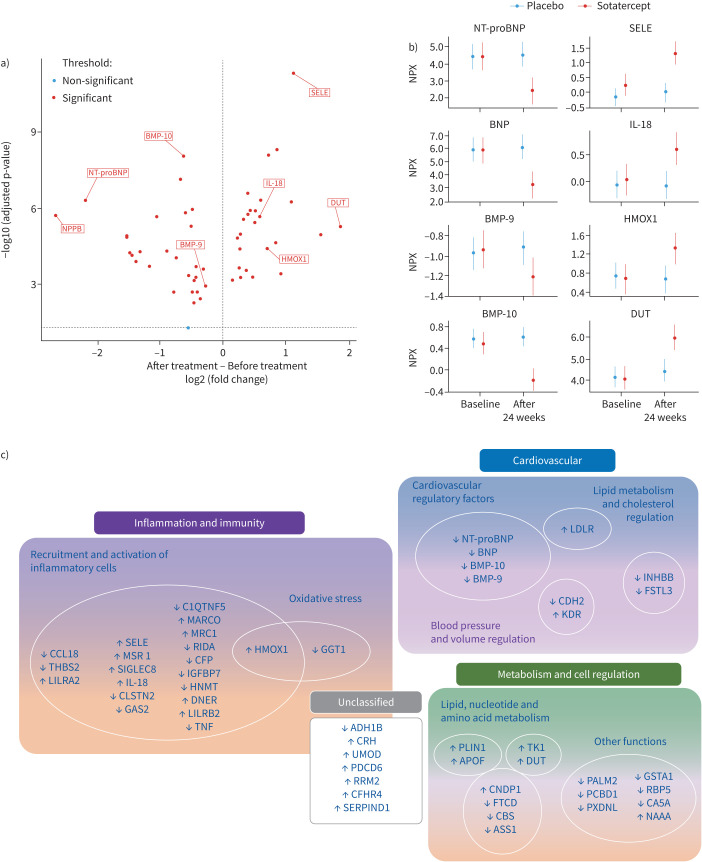
Analysis of differentially expressed proteins in pulmonary arterial hypertension (PAH) patients treated with sotatercept and placebo. a) Volcano plot illustrating differentially expressed proteins between baseline and week 24, comparing sotatercept and placebo groups. Each point represents a protein, with log_2_ (fold change) on the *x*-axis and statistical significance (−log_10_ (adjusted p-value)) on the *y*-axis. Significantly regulated proteins are highlighted in red. b) Point range plots highlight significant changes in proteins identified in the interaction term for the linear effects model. Each point represents the estimated mean value per group, with the vertical bars showing the 95% confidence intervals. NPX (Normalised Protein Expression), Olink's arbitrary unit, is represented on a Log_2_ scale. c) Biomarkers with significant differential expression changes between baseline and week 24, comparing sotatercept and placebo groups, are categorised based on their primary biological functions. BNP: brain natriuretic peptide; NT-proBNP: N-terminal pro-brain natriuretic peptide; SELE: E-selectin; IL: interleukin; BMP: bone morphogenetic protein; HMOX1: haem oxygenase 1; DUT: deoxyuridine triphosphatase.

This study is the first to analyse the effect of sotatercept on circulating biomarkers using large-scale proteomics. Our data demonstrated that sotatercept effectively blocks activins and significantly reduces other key members of the BMP/TGF-β family, including BMP-9 and BMP-10. These results confirm previous experimental studies on sotatercept's affinity for these ligands [1, 8]. BMP-9 and BMP-10 are implicated in hereditary haemorrhagic telangiectasia, a condition characterised by vessel dilatation and telangiectasia [5, 6]. We observed similar phenomena in PAH patients treated with sotatercept. This raises questions, especially since BMP-9-deficient rodents, with or without BMP-10 loss, resemble hereditary haemorrhagic telangiectasia more than PAH and are protected against pulmonary hypertension models [9–12]. The impact of sotatercept-induced reductions in BMP-9 and BMP-10 and its potential role in side effects like telangiectasia warrant further investigation.

We observed substantial changes in the levels of several inflammatory mediators, including E-selectin, IL-18, CCL18, and MARCO, which are involved in cell recruitment and activation. Additionally, there was an increase in HMOX1 and a decrease in GGT1, both crucial for oxidative stress. It is challenging to determine whether these changes indicate a reduction in oxidative stress and inflammation due to sotatercept or an activation, as seen with anti-PD1 agents. Further studies are needed to elucidate the role of sotatercept in modulating inflammation in acute and chronic models. Initial animal studies of pulmonary hypertension suggest that the sotatercept analogue reduces inflammatory recruitment, but the underlying mechanisms are not fully understood [7]. We also observed modulation of two distinct sets of differentially expressed proteins with clear roles in the cardiovascular system and cell metabolism following sotatercept treatment. These findings align with the understanding that dysregulation of cellular metabolism, particularly involving lipids, nucleotides, and amino acids, contributes to PAH progression. Gene set enrichment analysis indicates that the observed changes favour a decrease in these processes with sotatercept use, consistent with recognised alterations and increased cell metabolism in PAH [13, 14].

This study is retrospective, and selection bias cannot be excluded given the small cohort size and the absence of certain PAH subtypes, such as scleroderma-associated PAH. Nevertheless, it is based on samples from patients in phase 2 and 3 trials, ensuring their quality. It is possible that observed changes in circulating blood may not fully reflect modifications in the lungs, or could be indirect or related to compensatory mechanisms. Furthermore, there was no separate validation cohort.

In conclusion, our study enhances the understanding of PAH treatment by highlighting proteomic changes associated with sotatercept. The identification of proteins, including the reduction of BMP-9 and BMP-10, and changes in inflammatory mediators, offers valuable insights into the complex pathways influenced by sotatercept and its potential mechanisms of action. These findings pave the way for future research to better understand how sotatercept affects disease mechanisms and patient outcomes.

## Shareable PDF

10.1183/13993003.01483-2024.Shareable1 This one-page PDF can be shared freely online.
Shareable PDF ERJ-01483-2024.Shareable

## References

[1] Guignabert C, Humbert M. Targeting transforming growth factor-beta receptors in pulmonary hypertension. Eur Respir J 2021; 57: 2002341. doi:10.1183/13993003.02341-2020 32817256

[2] Humbert M, Sitbon O, Guignabert C, et al. Treatment of pulmonary arterial hypertension: recent progress and a look to the future. Lancet Respir Med 2023; 11: 804–819. doi:10.1016/S2213-2600(23)00264-3 37591298

[3] Hoeper MM, Badesch DB, Ghofrani HA, et al. Phase 3 trial of sotatercept for treatment of pulmonary arterial hypertension. N Engl J Med 2023; 388: 1478–1490. doi:10.1056/NEJMoa2213558 36877098

[4] Humbert M, McLaughlin V, Gibbs JSR, et al. Sotatercept for the treatment of pulmonary arterial hypertension. N Engl J Med 2021; 384: 1204–1215. doi:10.1056/NEJMoa2024277 33789009

[5] Ryanto GRT, Ikeda K, Miyagawa K, et al. An endothelial activin A-bone morphogenetic protein receptor type 2 link is overdriven in pulmonary hypertension. Nat Commun 2021; 12: 1720. doi:10.1038/s41467-021-21961-3 33741934 PMC7979873

[6] Joshi SR, Liu J, Bloom T, et al. Sotatercept analog suppresses inflammation to reverse experimental pulmonary arterial hypertension. Sci Rep 2022; 12: 7803. doi:10.1038/s41598-022-11435-x 35551212 PMC9098455

[7] Yung LM, Yang P, Joshi S, et al. ACTRIIA-Fc rebalances activin/GDF *versus* BMP signaling in pulmonary hypertension. Sci Transl Med 2020; 12: eaaz5660. doi:10.1126/scitranslmed.aaz5660 32404506 PMC8259900

[8] Aykul S, Martinez-Hackert E. Transforming growth factor-beta family ligands can function as antagonists by competing for type II receptor binding. J Biol Chem 2016; 291: 10792–10804. doi:10.1074/jbc.M115.713487 26961869 PMC4865925

[9] Bouvard C, Tu L, Rossi M, et al. Different cardiovascular and pulmonary phenotypes for single- and double-knock-out mice deficient in BMP9 and BMP10. Cardiovasc Res 2022; 118: 1805–1820. doi:10.1093/cvr/cvab187 34086873 PMC9215199

[10] Tu L, Desroches-Castan A, Mallet C, et al. Selective BMP-9 inhibition partially protects against experimental pulmonary hypertension. Circ Res 2019; 124: 846–855. doi:10.1161/CIRCRESAHA.118.313356 30636542

[11] Robert F, Certain MC, Baron A, et al. Disrupted BMP-9 signaling impairs pulmonary vascular integrity in hepatopulmonary syndrome. Am J Respir Crit Care Med 2024; 210: 648–661. doi:10.1164/rccm.202307-1289OC 38626313

[12] Berrebeh N, Mbouamboua Y, Thuillet R, et al. Bone morphogenetic protein-9 controls pulmonary vascular growth and remodeling. medRxiv 2023; preprint [10.1101/2023.06.02.23290910]. PMC12232436 40549904

[13] Humbert M, Guignabert C, Bonnet S, et al. Pathology and pathobiology of pulmonary hypertension: state of the art and research perspectives. Eur Respir J 2019; 53: 1801887. doi:10.1183/13993003.01887-2018 30545970 PMC6351340

[14] Guignabert C, Aman J, Bonnet S, et al. Pathology and pathobiology of pulmonary hypertension: current insights and future directions. Eur Respir J 2024; 64: 2401095. 10.1183/13993003.01095-2024 PMC11533988 39209474

