# Supplementary material for: Effect of sotatercept on circulating proteomics in pulmonary arterial hypertension
Source: Eur Respir J. 2024 Oct 31;64(4):2401483. doi: 10.1183/13993003.01483-2024 (PMC11525346; doi:10.1183/13993003.01483-2024)
Supplement: Supplementary file 1 [file ERJ-01483-2024.Shareable.pdf]

SHAREABLE PDF

Effect of sotatercept on circulating proteomics in pulmonary arterial hypertension

Laurent Savale [1,2,3], Ly Tu [1,2], Corinne Normand[1,2], Athénaïs Boucly [1,2,3], Olivier Sitbon [1,2,3], David Montani [1,2,3], Karen M. Olsson[4], Da-Hee Park[4], Jan Fuge [4], Jan C. Kamp [4], Marc Humbert [1,2,3], Marius M. Hoeper [4] and Christophe Guignabert [1,2]

[1]Université Paris-Saclay, Hypertension Pulmonaire: Physiopathology and Innovation Thérapeutique, HPPIT, Faculté de Médecine, Le Kremlin-Bicêtre, France. [2]INSERM UMR_S 999, HPPIT, Le Kremlin-Bicêtre, France. [3]Department of Respiratory and Intensive Care Medicine, Assistance Publique Hôpitaux de Paris, Hôpital Bicêtre, ERN-LUNG, Le Kremlin-Bicêtre, France. [4]Department for Respiratory Medicine and Infectious Diseases and German Centre of Lung Research (DZL), Hannover Medical School, Hannover, Germany.

Corresponding author: Christophe Guignabert (christophe.guignabert@inserm.fr)

Check for updates

Shareable abstract (@ERSpublications)
Proteomic analysis of circulating biomarkers reveals that sotatercept's impact extends beyond activins to influence BMP-9 and BMP-10, along with essential metabolic and inflammatory factors https://bit.ly/3Z5AZJ3

Cite this article as: Savale L, Tu L, Normand C, *et al*. Effect of sotatercept on circulating proteomics in pulmonary arterial hypertension. *Eur Respir J* 2024; 64: 2401483 [DOI: 10.1183/13993003.01483-2024].

This extracted version can be shared freely online.

Copyright ©The authors 2024.

This version is distributed under the terms of the Creative Commons Attribution Licence 4.0.

Received: 17 April 2024
Accepted: 20 Aug 2024

To the Editor:

Alterations in specific signalling pathways within the bone morphogenetic protein/transforming growth factor-β (BMP/TGF-β) family, involving several precisely regulated activator or inhibitor ligands, have been identified as pathogenic drivers of pulmonary arterial hypertension (PAH). These alterations, particularly affecting BMPRII and activin-dependent pathways, have led to innovative therapies, notably the development of sotatercept [1, 2]. Sotatercept, a fusion protein of the extracellular domain of human ACTRIIA and the Fc domain of human IgG1, has shown promising results in improving key clinical, functional, and haemodynamic parameters in PAH patients, as evidenced by positive results in the phase 2 PULSAR and phase 3 STELLAR trials [3, 4]. This progress was partly based on preclinical studies showing that reducing activin-induced Smad2/3 phosphorylation levels, by suppressing activin production in mice [5] or using soluble receptors in rats [6, 7], can attenuate pulmonary vascular remodelling. Despite these advancements, the precise mechanisms of action of these approaches in humans and rodents need to be better understood to enhance these valuable tools. Sotatercept raises several critical questions regarding its mechanism of action, and a deeper understanding could reveal the pathophysiological mechanisms of PAH, leading to more effective therapeutic approaches.

CC BY
